# Precursor fractions of neurotensin and enkephalin might point to molecular mechanisms of cancer risk modulation during a lifestyle-intervention in germline *BRCA1/2* gene mutation carriers

**DOI:** 10.1007/s10549-020-06070-x

**Published:** 2021-02-04

**Authors:** Sabine Grill, Maryam Yahiaoui-Doktor, Maryam Basrai, Joachim Struck, Janin Schulte, Anika Berling-Ernst, Christoph Engel, Mirjam Ullrich, Jacqueline Lammert, Stephan C. Bischoff, Thorsten Schmidt, Uwe Niederberger, Dimitrios Chronas, Kerstin Rhiem, Rita Schmutzler, Martin Halle, Marion Kiechle

**Affiliations:** 1 Department of Gynecology and Center for Hereditary Breast and Ovarian Cancer, Klinikum Rechts Der Isar, Technical University Munich (TUM), Munich, Germany; 2 Institute for Medical Informatics, Statistics and Epidemiology (IMISE), University of Leipzig, Leipzig, Germany; 3 Department of Prevention, Rehabilitation and Sports Medicine, Faculty of Medicine, University Hospital Rechts Der Isar, Technical University of Munich (TUM), Munich, Germany; 4 Institute of Nutritional Medicine, University of Hohenheim, Stuttgart, Germany; 5 Comprehensive Cancer Center, University Hospital Schleswig-Holstein, Kiel, Germany; 6 Institute for Medical Psychology and Medical Sociology, University Hospital Schleswig-Holstein, Kiel, Germany; 7 Center for Hereditary Breast and Ovarian Cancer, University Hospital Cologne, Cologne, Germany; 8 SphingoTec GmbH, Hennigsdorf, Germany

**Keywords:** *BRCA1/2* mutation carriers, Lifestyle intervention, Physical activity, Cardiopulmonary fitness, Nutrition, Breast cancer

## Abstract

**Background:**

Germline *BRCA1/2* mutation carriers (gBMC) face increased cancer risks that are modulated via non-genetic lifestyle factors whose underlying molecular mechanisms are unknown. The peptides Neurotensin (NT) and Enkephalin (ENK)—involved in tumorigenesis and obesity-related diseases—are of interest. We wanted to know whether these biomarkers differ between gBMC and women from the general population and what effect a 1-year lifestyle-intervention has in gBMC.

**Methods:**

The stable precursor fragments pro-NT and pro-ENK were measured at study entry (SE), after 3 and 12 months for 68 women from LIBRE-1 (a controlled lifestyle-intervention feasibility trial for gBMC involving structured endurance training and the Mediterranean Diet). The SE values were compared with a cohort of the general population including female subjects with and without previous cancer disease, non-suggestive for hereditary breast and ovarian cancer (OMA-reference). For LIBRE-1, we analysed the association between the intervention-related change in the two biomarkers and certain lifestyle factors.

**Results:**

At SE, gBMC had a higher median pro-NT than OMA-reference (in the subgroups with previous cancer 117 vs. 91 pmol/L, *p* = 0.002). Non-diseased gBMC had lower median pro-ENK levels when compared to the non-diseased reference group. VO2peak and pro-NT 1-year change in LIBRE-1 were inversely correlated (*r* = − 0.435; CI − 0.653 to − 0.151; *p* = 0.004). Pro-ENK correlated positively with VO2peak at SE (*r* = 0.323; CI 0.061–0.544; *p* = 0.017). Regression analyses showed an inverse association of 1-year changes for pro-NT and Omega-6/Omega-3 (Estimate: − 37.9, *p* = 0.097/0.080) in multivariate analysis.

**Conclusion:**

Our results give first indications for lifestyle-related modification particularly of pro-NT in gBMC.

## Background

Lifestyle factors such as physical inactivity, obesity and hypercaloric nutrition have been shown to substantially impact breast cancer (BC) risk [1–7]. To what extent modifiable lifestyle factors affect cancer risk in women genetically at high risk of developing BC, such as carriers of a germline mutation in the genes *BRCA1* and *BRCA2 (*g*BRCA1/2)* has been far less widely researched. Of note is that the penetrance of certain inherited *BRCA1* mutations has been increasing 24% to 67% as generations proceed [8–10]. Amongst other reasons this increase might be attributed to an alteration of the social and lifestyle environment [8, 11].

In light of this the LIBRE-1 trial, a prospective lifestyle-intervention randomized controlled trial, was initiated to investigate whether a structured 1-year intervention program ameliorates cardiopulmonary fitness, body mass index (BMI) and the nutritional pattern in female *gBRCA1/2* mutation carriers [12–14]. Additionally, the identification of lifestyle-related biomarkers for molecular mechanisms that are involved in tumorigenesis in g*BRCA1/2* mutation carriers is important and therefore certain promising biomarkers were measured in the LIBRE-1 trial. Two of these, which are the focus of this work: neurotensin (NT) and enkephalin (ENK) have been identified as markers linking cardiovascular, cardiometabolic and cancer risk.

### Neurotensin/pro-neurotensin (pro-NT)

Neurotensin (NT) is a 13-amino acid peptide, which is mainly found in the central nervous system and in the small intestine. It behaves as a neuromodulator in the brain by regulating the anorectic effect and is an important factor in nutrient metabolism, particularly through lipid ingestion and fat storage [15–19]. Clinical studies indicate that increased levels of peripheral NT occur in obesity and are directly linked to features of insulin resistance in humans [17, 19–21]. Within the Framingham Heart Study, NT was associated with incident major cardiovascular events, independently of traditional cardiovascular risk factors [22]. Melander et al. conducted a large cohort study, which confirmed a contribution of NT to obesity-derived diseases such as cardiovascular and in particular BC [21]. Recently, Li and colleagues were able to demonstrate a protection from obesity in NT-deficient mice fed with a high-fat diet [17].

### Enkephalin/pro-enkephalin (pro-ENK)

As part of the endogenous opioid peptide system, Methionine-ENK (MetENK), is deemed to be of considerable importance in tumorigenesis by averting tumor cell proliferation [23–27]. Within the framework of two Swedish cohort studies, fasting plasma of 3498 non-diseased women was examined to quantify pro-ENK, the stable precursor of ENK, and predict its impact on BC risk [28]. After a median follow-up of 14.7 years, women with pro-ENK values in the lowest quartile were found to be three to five times more susceptible to BC than women with pro-ENK values in the highest quartile [28, 29]. Intriguingly, more recent findings indicate a favourable effect of MetENK concerning obesity and its related diseases [30]. Cardiovascular function has also been found to be affected by this biomarker and seems to be modulated by physical activity [31–35].

Within an experimental study, the effect of weekly moderate-intensity exercise training on the activation of the cardiac opioid system of a diet-induced obesity model in rats was compared [36]. While sedentary animals fed with a high fat diet presented lower cardiac pro-ENK messenger ribonucleic acid (mRNA) levels, a level-dependent activation of the cardiac opioid system was seen in the active group [36]. This could be relevant in the context of our study, as there is emerging evidence on an association between *BRCA1/2* deficiency and various cardiovascular disorders such as atherosclerosis and ischemic heart disease [37]. Certainly, premature menopause as a consequence of prophylactic bilateral salpingo-oophorectomy (PBSO) and /or potential cardiotoxic effects of cancer treatment (chemotherapy/radiotherapy in case of breast cancer) may have a decisive impact. Recently, a causal part for the genes *BRCA1* and *BRCA2* in cardiovascular diseases was described [37]. The role of these two genes as gatekeepers in cardiac function and structure can increase susceptibility to cardiac damage when their function is compromised [38].

We, therefore, wanted to answer two specific questions: i) whether g*BRCA1/2* mutation carriers differ from women of the general population, that are not indicative for hereditary breast and ovarian cancer regarding plasma concentration of NT and ENK, ii) what is the effect of a 1-year lifestyle-intervention on NT and ENK plasma levels in g*BRCA1/2* mutation carriers.

## Methods

### Study cohorts and patient recruitment

The LIBRE-1 trial is the feasibility phase of a multicenter, prospective, two-armed, randomized (1:1) and controlled lifestyle-intervention trial that enrolled 68 women with and without a previous BC or ovarian cancer (OC), aged 18–72 years, with a germline mutation in the *BRCA1* or *BRCA2* gene. From February to July 2014, study participants were recruited at three of the study centres of the German Consortium of Hereditary Breast and Ovarian Cancer (www.konsortium-familiaerer-brustkrebs.de), Cologne, Kiel and Munich. The study protocol has been published in detail elsewhere [12, 13]. The LIBRE-1 trial was approved by the ethics committee of all participating centres and written informed consent was obtained from all participants prior to study entry (SE, baseline).

**Fig. 1:**
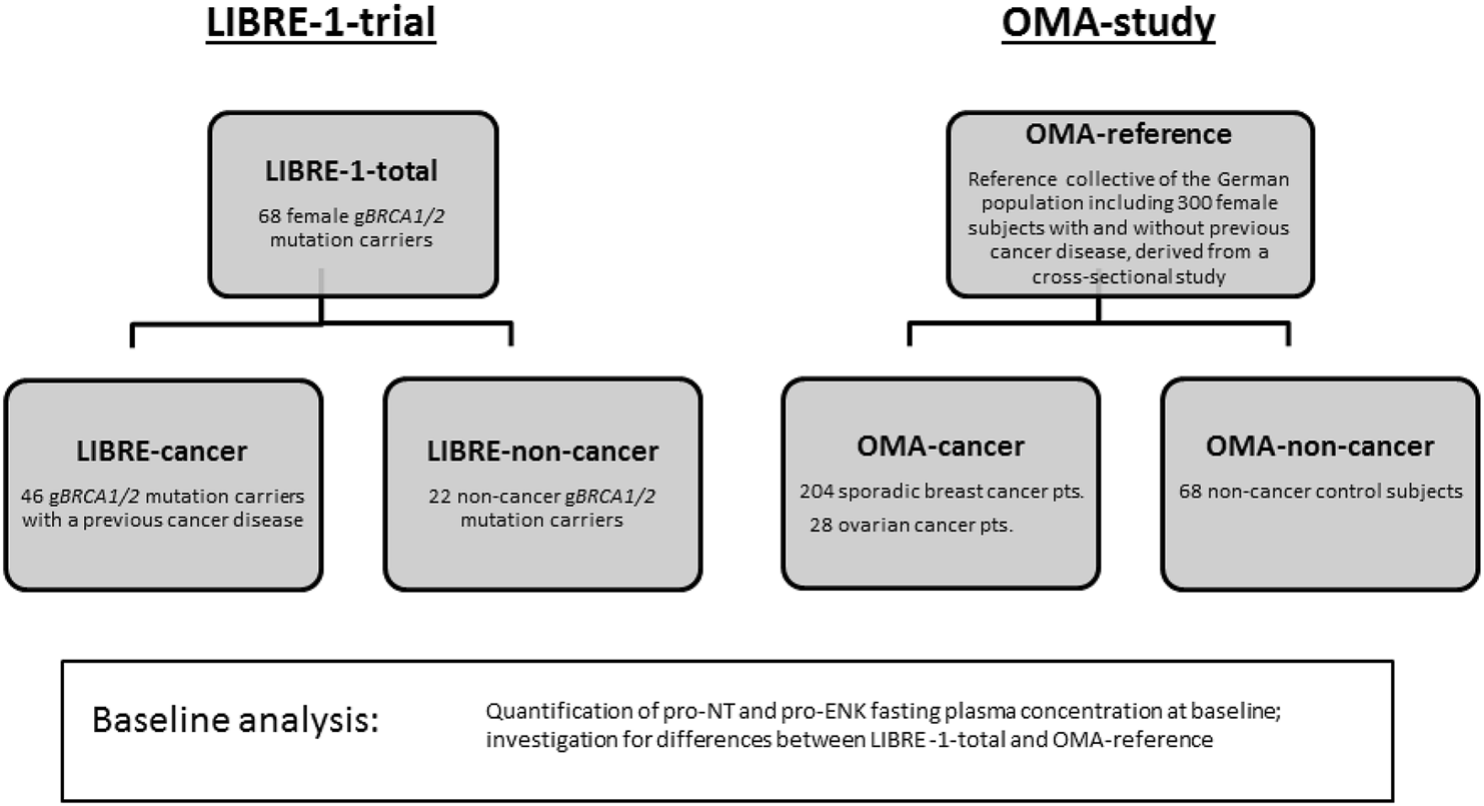
Schematic overview of the study population and baseline analysis. At baseline, pro-NT and pro-ENK fasting plasma concentrations were quantified within the two collectives, LIBRE-1-total including *BRCA1/2* mutation carriers with and without previous cancer disease derived from the LIBRE-1-pilot trial and the OMA-reference, which encompasses women of the general population with and without previous cancer disease respectively, not indicative for hereditary breast and ovarian cancer. Baseline levels were then compared

In order to answer our first question, we also used data from a cohort of the general population including female subjects with and without previous cancer disease, which were involved in a cross-sectional study (OMA-reference). The OMA study had previously been conducted at the Department of Gynecology and Obstetrics of the Technical University Munich and the Red Cross Women´s hospital in Munich and enrolled 204 BC patients, 28 OC patients and 68 control subjects without previous or current cancer [16, 29] (Fig. 1).

### Immunoassays

As mature NT and ENK are unstable both in vitro and in vivo [21, 28, 39], the fasting plasma concentration of pro-NT, a stable 117-amino acid fragment from the NT precursor hormone and stoichiometrically equal to NT, as well as pro-ENK considered a stable 41-amino-acid fragment of the ENK precursor hormone following a 12-h fasting period was measured instead.

For OMA-reference pro-NT and pro-ENK fasting plasma levels were measured just once at SE, while this was done at SE as well as subsequent time points [plus 3 months (V1) and plus 12 months (V2)] for LIBRE-1-total. The plasma aliquots of LIBRE-1-total as well as OMA-reference were immediately frozen after separation and stored at − 80 °C.

#### Pro-neurotensin (pro-NT)

Chemiluminometric sandwich immunoassay was applied to quantify the pro-NT precursor fragment (proNT 1–117), a stable fragment of the NT precursor hormone (SphingoTec GmbH, Hennigsdorf, Germany) [21]. The assay was modified using two mouse monoclonal antibodies against pro-NT 8-25 and pro-NT 44-62, native human pro-NT diluted in horse serum (Sigma-Aldrich, Germany, Munich) for calibration and detecting signals in a microplate luminometer. The functional assay sensitivity was 3 pmol/L, defined as the lowest measurement value detectable with a precision of maximum 20% inter-assay coefficient of variance (CV). The mean inter-assay CV was 3.7% in the measuring range 3–270 pmol/L.

#### Pro-enkephalin A 119–159 (pro-ENK)

Pro-ENK was quantified using a similar microtiter plate-based sandwich immunoassay approach (SphingoTec GmbH, Hennigsdorf, Germany), which has been published previously [40]. Two mouse monoclonal anti-pro-ENK antibodies were developed by immunizing mice with pro-ENK peptide consisting of amino acids 119–159 of pro-ENK [40]. The detection limit was stated to be 7 pmol/L and the mean inter-assay CV yielded 5.7% in the measuring range 10.9–686.3 pmol/L.

### Lifestyle-intervention

**Fig. 2:**
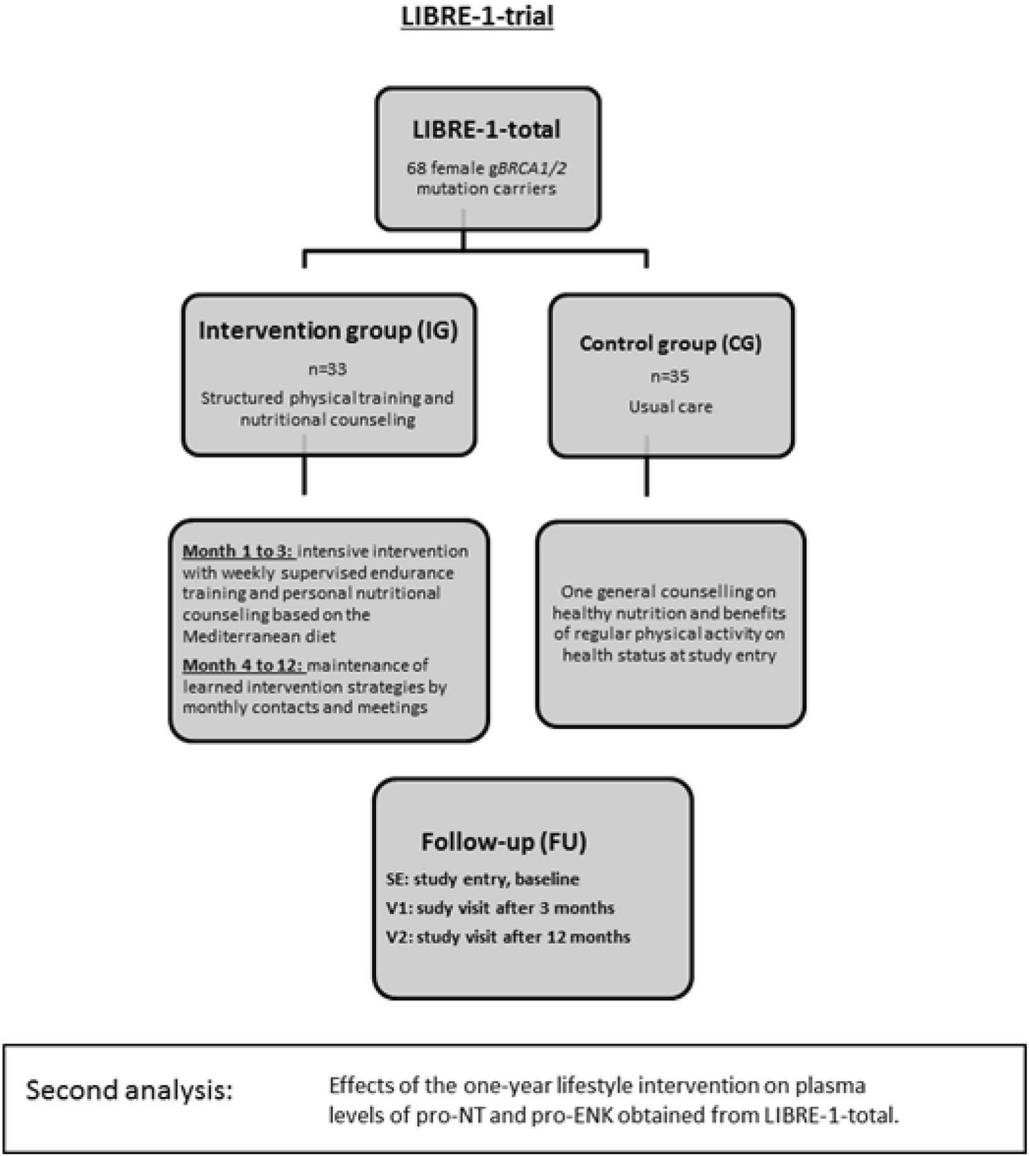
Schematic overview of second analysis: effects of the 1-year lifestyle-intervention on plasma levels of pro-NT and pro-ENK in LIBRE-1-total. Within a second analysis, the effect of a 1-year lifestyle-intervention on pro-NT and pro-ENK plasma levels in g*BRCA1/2* mutation carriers of the LIBRE-1-trial was investigated

We considered the effects of the 1-year lifestyle-intervention on plasma levels of pro-NT and pro-ENK in LIBRE-1-total only, as OMA-reference only had one measurement available (Fig. 2).

Intervention included structured endurance training in addition to nutrition education based upon the concept of the Mediterranean Diet (MedD) [12–14]. The principal aspects of the MedD encompass high consumption of olive oil, legumes, unrefined cereals, fruits and vegetables, moderate consumption of seafood and dairy products, and reduced intake of meat products and sweets [41, 42]. The intervention group (IG) received structured and supervised training sessions within the first three months (SE to V1), considered as the intense phase of the intervention program, aiming a performance target of ≥ 18 MET*h/week (MET = metabolic equivalent task) [12–14]. Their program also comprised regular nutritional group education on the MedD [12–14]. During the subsequent nine months (to V2), participants of the IG were encouraged to complete exercise units as home-based training thrice weekly. Group training sessions continued at monthly intervals, accompanied by regular telephone contact as motivation. Compared with this, the control group (CG) attended only one lecture on physical activity and the benefits of healthy nutrition [12–14]. To objectively assess the impact on cardiopulmonary fitness, cardiopulmonary exercise testing (CPET) was performed at SE, V1 and V2, which measures the respiratory gases under exercise stress and thus captures maximal oxygen uptake [VO2peak (mL/min/kg)], as well as aerobic and anaerobic respiratory capacity [14]. Target parameters were defined as VO2peak as well as VO_2_ at ventilatory aerobic and anaerobic thresholds to give information on the cardiopulmonary fitness level at the specified time points of the study [14, 43].

Furthermore, at each time point, a clinical examination was conducted to assess anthropometric data (age, BMI, waist circumference), medical history, social background and risk factors for carcinogenesis for all participants. Blood samples were also collected, in order to determine various blood values. To record dietary behaviour, Omega-3, Omega-6, and Omega-9 fatty acid (FA) content in the membrane of erythrocytes was assessed [44].

### Statistical analysis

We first described the LIBRE-1-total participants and then compared their fasting plasma levels of pro-NT and pro-ENK (and Age and BMI) with the OMA-reference participants. We then tested pairwise whether there were statistical differences between g*BRCA1/2* mutations carriers with or without a prior diagnosis of cancer (LIBRE-cancer; LIBRE-non-cancer) and the OMA-reference (OMA-non-cancer and OMA-cancer). For this, we applied the Mann–Whitney *U* test.

In a second step, we focused on the LIBRE-1-total participants. We were interested in the effects of the lifestyle-intervention in LIBRE-1 on pro-NT and pro-ENK plasma levels. We first determined the Pearson’s correlation coefficient between the two biomarkers and VO2peak at each study time point. Then, we looked at the changes (SE to V2) in pro-NT and pro-ENK in the LIBRE-1-total in univariate and multivariate linear regression analyses. The explanatory variables were: BMI change, age at baseline, g*BRCA1/2* mutation status, VO2peak change for cardiopulmonary fitness, anti-inflammatory fatty acid (FA) profile in erythrocyte membrane (ratio of Omega-6 and Omega-3 FA) change as an objective assessment of dietary behavior, as well as the study arm and whether the participant had previously had BC or OC [12].

All statistical analysis was carried out using R version 3.6.3 (The R Foundation for Statistical Computing, Vienna, Austria) in the R-Studio environment version 1.2.5001 (Boston, Massachusetts, USA). Statistical significance in this study was chosen as *p* ≤ 0.05.

## Results

### Study participants

The baseline characteristics of the LIBRE-1-total are described in Table 1. Of the 68 participants of the LIBRE-1-total, 33 were randomized to the IG of which 23 had previously suffered from BC or OC disease. Of the 35 g*BRCA1/2* mutation carriers randomized to the CG, 23 had a previous BC/OC disease.

**Table 1:** Patient characteristics at study entry in the LIBRE-1-total

	Intervention group	Control group	Total
	*n* = 33	*n* = 35	*n* = 68
Cancer	23 (69.7%)	23 (65.7%)	46 (67.6%)
Breast	21 (63.6%)	22 (62.8%)	43 (63.2%)
Ovarian	1 (3%)	1 (2.8%)	2 (2.9%)
Both	1 (3%)	0	1 (1.5%)
g*BRCA1*	24 (72.7%)	18 (51.4%)	42 (61.7%)
Age (years)^a^	42 (27–72)	42 (24–68)	42 (24–72)
BMI (kg/m^2^)^a^	22.2 (18.0–45.5)	23.6 (18.3–42.2)	23.2 (18.0–45.4)
VO2peak (ml/min/kg)^a^	24 (12–42)	28 (15–38)	26 (12–42)

Patient characteristics at study entry in the LIBRE-1-total stratified by intervention arm

^a^Median (IQR)

The median age of the LIBRE-1-total participants was 42 years. Forty-two (61.8%) of the participants were carriers of a g*BRCA1* mutation, and a total of 46 (67.6%) had previously had cancer disease: 43 cases of BC and another three cases of OC. In terms of preventive options, 21 (30.9%) of the mutation carriers had undergone a mastectomy (bilateral mastectomy *n* = 14; unilateral mastectomy *n* = 7), whereas 31 (45.6%) of the participating women had received PBSO.

### Baseline data compared to OMA-reference, a reference group of the general population

The results of the comparison of the LIBRE-1-total and the OMA-reference group are illustrated in Table 2. The subgroup OMA-cancer had the highest median age of 59 years (IQR: 50–68), which was statistically significant in comparison to all 3 other groups (all *p* < 0.001).

**Table 2:** Comparison between LIBRE-1-total and OMA-reference stratified by disease status at study entry

	Non-diseased (*n* = 90)		Previous cancer disease (*n* = 268)		All (*n* = 368)
	LIBRE-non-cancer (*n* = 22)	OMA-non-cancer (*n* = 68)	LIBRE-cancer (*n* = 46)	OMA-cancer (*n* = 232)	
Age (years)^a^	41 (34–45)	43 (33–53)	43 (35–49)	59 (50–68)	52 (44–64)
BMI (kg/m^2^)^a^	25 (22–32)	23 (20–26)	23 (21–27)	24 (22–27)	24 (21–26)
pro-NT (pmol/L)^a^	112 (86–146)	94 (64–133)	117(89–140)	91 (65–118)	96 (69–129)
pro-ENK (pmol/L)^a^	61 (54–73)	70 (60–80)	69 (62–76)	63 (54–75)	65 (56–76)

Age, BMI as well as pro-NT and pro-ENK plasma levels were investigated in the LIBRE-1-total and OMA-reference, respectively. A comparison was then made and additional substratification by disease status was performed

^a^Median (IQR)

LIBRE-1-total had higher median concentrations of pro-NT, which was statistically significant in the pair-wise comparison of the OMA-cancer and LIBRE-cancer subgroups (117 pmol/L vs. 91 pmol/L, *p* = 0.002). For pro-ENK, the highest absolute difference was between LIBRE-non-cancer and OMA-non-cancer, yet not significant (61 pmol/L vs. 70 pmol/L, *p* = 0.158). OMA-cancer had lower median pro-ENK levels than LIBRE-cancer (63 vs. 69 pmol/L, *p* = 0.045), and OMA-non-cancer higher than OMA-cancer (70 vs. 63 pmol/L, *p* = 0.046)*.* All other differences were not statistically significant.

### Association of physical activity with plasma levels of pro-NT and pro-ENK (LIBRE-1-total)

**Fig. 3:**
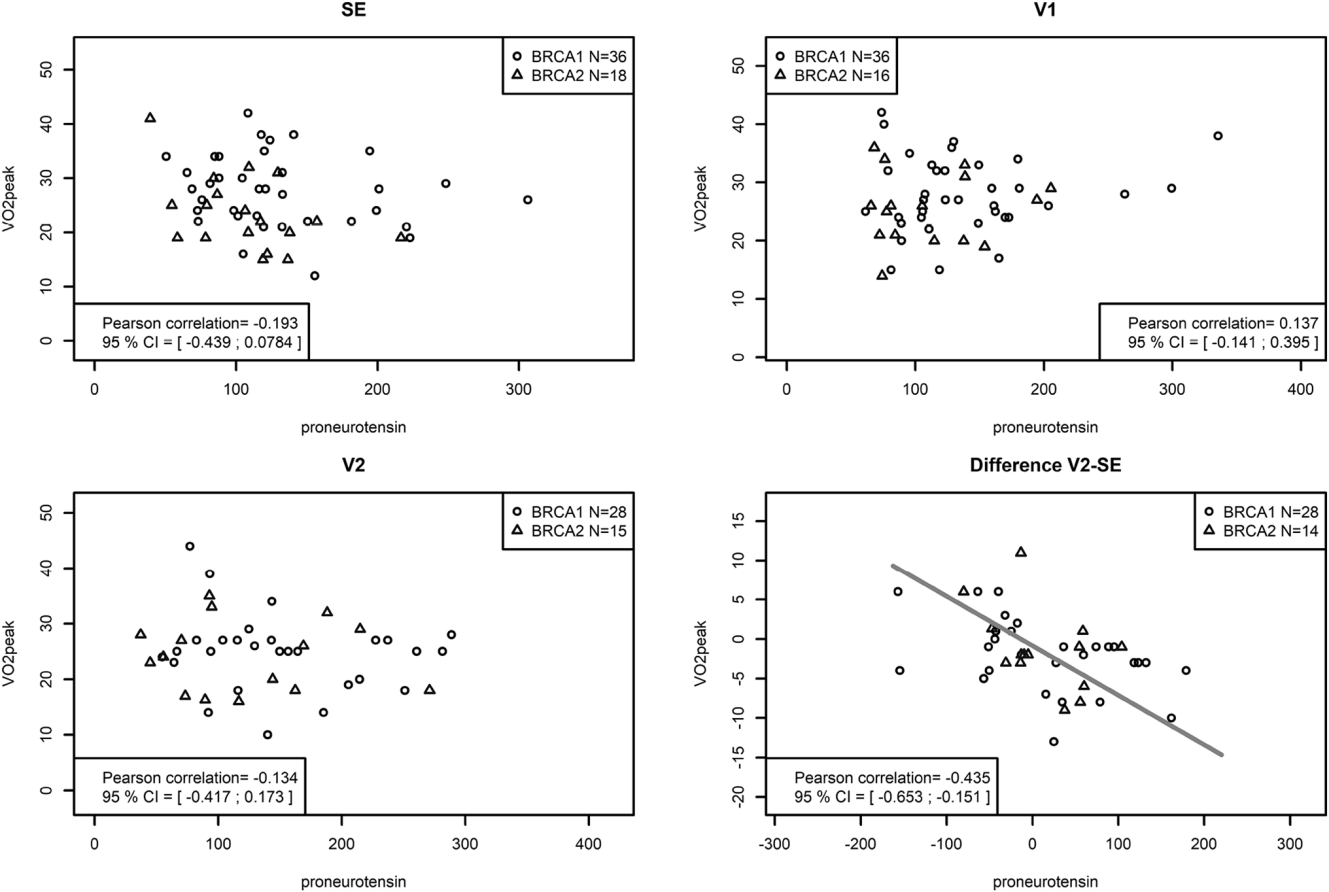
VO2 peak depending correlation to pro-NT plasma levels at time points SE, V1, V2 and difference V2-SE. It shows the association between physical activity with plasma levels of pro-NT in LIBRE-1-total at time points SE, V1, V2 and difference V2-SE. VO2peak changes over the 1-year intervention were inversely correlated with changes in pro-NT (*r* = − 0.435; CI − 0.653 to − 0.151; *p* = 0.004)

**Fig. 4:**
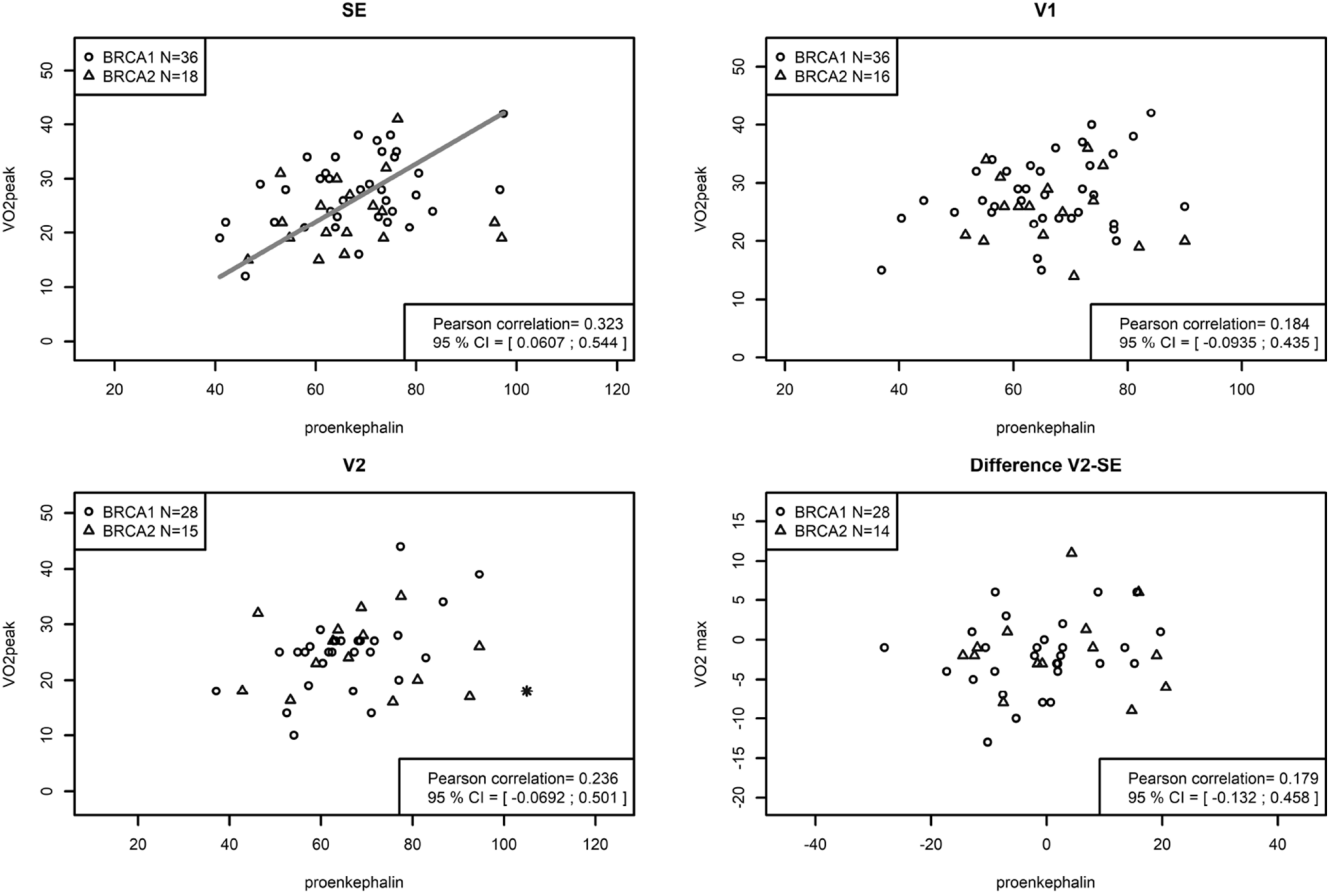
VO2 peak depending correlation to pro-ENK plasma levels at time points SE, V1, V2 and difference V2-SE. It presents the association between physical activity with plasma levels of pro-ENK in LIBRE-1-total at time points SE, V1, V2 and difference V2-SE. VO2peak and pro-ENK showed a positive correlation at SE only (*r* = 0.323; CI 0.061–0.544; *p* = 0.017)

VO2peak showed a weak negative association with pro-NT and positive association with pro-ENK biomarkers, albeit at different time points. VO2peak changes over the 1-year intervention were inversely correlated with changes in pro-NT (*r* = − 0.435; CI − 0.653 to − 0.151; *p* = 0.004) (Fig. 3), while VO2peak and pro-ENK showed a positive correlation at SE only (*r* = 0.323; CI 0.061–0.544; *p* = 0.017) (Fig. 4).

### Effects of the 1-year lifestyle-intervention on pro-NT and pro-ENK plasma levels (LIBRE-1-total)

In both the univariate and multivariate analysis, VO2peak was associated statistically significant with a reduction in pro-NT levels (Estimate: − 6.9 and − 6.8, *p* = 0.004 and 0.005, respectively) (Table 3). A consistent, yet non-significant inverse association with the ratio of Omega 6/Omega 3 FA in the erythrocyte membrane (Estimate: − 32.7 and − 37.9, *p* = 0.097 and 0.080 respectively) was shown (Table 3). Age had a consistent, non-significant association (Estimate: − 1.3, *p* = 0.190 and 0.312 respectively) (Table 3).

**Table 3:** Linear regression analysis for pro-NT (difference of V2-SE, *n* = 55)

	Univariate analysis		Multivariate analysis	
	Estimate (95% CI)	*p* value	Estimate (95% CI)	*p* value
Diff. in BMI V2-SE (kg/m^2^)	− 6.4 (− 20.0 to 7.2)	0.347	− 2.3 (− 17.5 to 12.9)	0.756
Age (years)	− 1.3 (− 3.3 to 0.7)	0.190	− 1.3 (− 3.8 to 1.3)	0.312
*gBRCA2* (*n*)	− 5.9 (− 51.0 to 39.2)	0.792	11.4 (− 38.0 to 60.8)	0.643
Diff. in VO2peak V2-SE (mL/kg/min)	− 6.9 (− 11.5 to − 2.4)	0.004*	− 6.8 (− 11.4 to – 2.2)	0.005*
Diff. in ratio: Omega6/Omega3 V2-SE	− 32.7 (− 71.5 to 6.2)	0.097	− 37.9 (− 80.7 to 4.8)	0.080
Study arm intervention	4.3 (− 38.4 to 47.0)	0.840	12.7 (− 31.9 to 57.3)	0.566
Previous BC/OC	19.3 (− 24.8 to 63.4)	0.383	14.2 (− 34.7 to 63.2)	0.558

Effects of the 1-year lifestyle-intervention on pro-NT plasma levels in LIBRE-1-total was investigated. In univariate and multivariate analysis, VO2peak was associated statistically significant with a reduction in pro-NT levels (Estimate: − 6.9 and − 6.8, *p* = 0.004 and 0.005, respectively). A consistent, yet non-significant inverse association with the ratio of Omega 6/Omega 3 FA in the erythrocyte membrane (Estimate: − 32.7 and − 37.9, *p* = 0.097 and 0.080, respectively) was shown

*Significant

In the case of pro-ENK, we found only age to have a statistically significant association, albeit only in univariate analysis (Estimate: 0.3, *p* = 0.037) (Table 4). VO2peak had a small positive and non-significant association with pro-ENK in uni-and multivariate analyses (Estimate: 0.4 and 0.3, *p* = 0.256 and 0.383) (Table 4). There was no association with BMI change over the 1-year intervention for pro-NT and pro-ENK (Tables 3, 4).

**Table 4:** Linear regression analysis for pro-ENK (difference of V2-SE, *n* = 55)

	Univariate analysis		Multivariate analysis	
	Estimate (95% CI)	*p* value	Estimate (95% CI)	*p* value
Diff. in BMI V2-SE (kg/m^2^)	0.9 (− 1.2 to 3.1)	0.379	− 0.1 (− 2.8 to 2.5)	0.925
Age (years)	0.3 (0.02–0.6)	0.037*	0.3 (− 0.2 to 0.7)	0.225
*gBRCA2 *(*n*)	3.0 (− 4.3 to 10.4)	0.413	1.7 (− 6.9 to 10.4)	0.684
Diff. in VO2peak V2-SE (mL/kg/min)	0.4 (− 0.3 to 1.2)	0.256	0.3 (− 0.5 to 1.1)	0.383
Diff. in ratio: Omega6/Omega3 V2-SE	− 2.4 (− 9.3 to 4.5)	0.480	− 3.6 (− 11.1 to 3.8)	0.332
Study arm intervention	− 2.7 (− 9.6 to 4.3)	0.447	− 1.9 (− 9.7 to 5.8)	0.615
Previous BC/OC	− 3.7 (− 11.0 to 3.5)	0.303	− 4.4 (− 13.0 to 4.1)	0.300

Effects of the 1-year lifestyle-intervention on pro-ENK plasma levels in LIBRE-1-total were evaluated performing univariate and multivariate analysis. For pro-ENK, we found only age to have a statistically significant association, albeit only in univariate analysis (Estimate: 0.3, *p* = 0.037). VO2peak had a small positive and non-significant association with pro-ENK in both sets of analyses (Estimate: 0.4 and 0.3, *p* = 0.256 and 0.383)

*Significant

## Discussion

We aimed to identify lifestyle-related biomarkers for molecular mechanisms that are involved in tumorigenesis in g*BRCA1/2* mutation carriers. NT and ENK are known to be linked with cardiovascular, cardiometabolic and cancer risk, and emerging evidence suggests direct associations with lifestyle factors [17, 19–21, 30, 33, 45]. We therefore aimed to investigate if NT and ENK plasma levels might be altered by a structured lifestyle-intervention program consisting of endurance training alongside the MedD in the context of g*BRCA1/2* mutation carriers, within the lifestyle-intervention trial LIBRE-1 [12–14].

Initially, we wanted to know whether g*BRCA1/2* mutation carriers inherently differ from women of the general population regarding NT and ENK fasting plasma concentrations. Therefore, plasma levels of the stable precursors of NT and ENK, pro-NT and pro-ENK were analyzed in the LIBRE-1-total, as well as the OMA-reference group at SE. The median pro-NT fasting plasma concentration was higher in LIBRE-1-total participants, reaching statistical significance in the comparison of LIBRE-cancer and OMA-cancer subgroups. Higher pro-NT levels would be expected with overweight or obesity, however the median BMI was comparable throughout our studied subgroups [17, 19, 20, 40]. The OMA-cancer subgroup was in median older than the others, however we found age not to be significantly associated with pro-NT within uni- and multivariate analyses. A large control collective from the Malmö cancer and diet (MDC) study of 1929 healthy subjects did not show a significant correlation between age and pro-NT, which points to an age-independent pro-NT activity [16]. This might point to an intrinsic disposition in the LIBRE-cancer subgroup given that pro-NT is in turn associated with elevated BC risk [21]. Discrepant results were obtained for pro-ENK. Participants of the LIBRE-non-cancer subgroup had lower median pro-ENK plasma concentrations compared to their OMA-non-cancer peers, which may indicate an adverse risk constellation for g*BRCA1/2* mutation carriers. Yet, compared with this, the subgroup LIBRE-cancer presented more favorable pro-ENK levels than participants of OMA-cancer, a difference that was marginally statistically significant (63 pmol/L vs. 69 pmol/L, *p* = 0.045). Univariate analysis showed a significant age-dependency, yet this disappeared in the multivariate analysis. Intriguingly, recent findings indicate a potential age–dependent increase of pro-ENK [46]. In contrast to these, the LIBRE-cancer subgroup had higher pro-ENK levels compared to the OMA-cancer subgroup, who were in median 16 years older. This might be explained by pre-existing stronger health awareness of the LIBRE-cancer subgroup, who for instance had a better cardiopulmonary fitness level compared to the LIBRE-non-cancer subgroup at SE, reflected by higher initial levels of VO2peak (mL/min/kg). A comparison with the cardiopulmonary fitness of the OMA-cancer subgroup is however not possible as CPET was not carried out in the OMA-reference study. Another influencing factor might be an endogenous compensation mechanism subsequent to cardiotoxic cancer therapy, especially when considering a possibly higher susceptibility to cardiac muscle disorder in g*BRCA1/2* mutation carriers [34]. However, further research is required to answer this question.

We then investigated the effect of a 1-year lifestyle-intervention on pro-NT and pro-ENK plasma levels in the g*BRCA1/2* mutation carriers within the lifestyle-intervention feasibility trial LIBRE-1. We saw a statistically significant inverse correlation between pro-NT plasma levels and VO2peak changes over the 1-year intervention (− 0.435; CI − 0.653 to − 0.151; *p* = 0.004), which also remained consistent in univariate and multivariate regression analyses. Based on previous reports from the LIBRE-1 trial [11, 12], which point out the over-motivation of the LIBRE-1 participants particularly in the CG, we consider VO2peak derived from CPET to be the most objective parameter for cardiopulmonary fitness.

Pro-ENK plasma concentration was positively correlated to increasing VO2peak at the study time point SE (0.323; CI 0.061–0.544; *p* = 0.017), which is in agreement with corresponding studies that postulated a relationship between physical training and plasma concentration of pro-ENK [47, 48]. Yet, this was not reproducible in follow-ups at V1 and V2, nor for the change through the 1-year lifestyle-intervention. Particularly, participants of LIBRE-cancer, who had the highest pro-ENK levels at SE and made up the majority of our study population, barely improved their pro-ENK plasma levels. A possible explanation might be that the impact of additional exercise sessions on proportionally elevated pro-ENK levels in LIBRE-cancer are less pronounced compared to LIBRE-non-cancer, who were physically less active at SE, reflected by lower proENK concentration. Existing evidence indicates a trainable response regarding pro-ENK concentrations [47, 48]. Kjær et al. described highest pro-ENK concentrations at 54% of the VO2peak in trained endurance athletes, however with further increasing exercise intensities the pro-ENK level decreased to resting concentrations [48]. Compared to this, the response patterns of untrained probands resulted in an increase in pro-ENK plasma concentrations with increasing exercise intensities [48]. The precise mechanism and explanation remains unknown, but this may be the emergence of a type of “adrenal exhaustion” due to a potential over-stimulation of the adrenal gland, from which pro-ENK is released [47].

We also built statistical models (linear regression) for pro-NT and pro-ENK plasma concentrations taking into consideration physical, clinical as well as nutritional parameters. Pro-NT showed a consistent, yet non-significant inverse association with the ratio of Omega 6/Omega 3 FA (an objective assessment of dietary patterns) in uni-and multivariate analysis (Estimate: − 32.7 and − 37.9, *p* = 0.097 and 0.080, respectively). These results are in agreement with previous findings in the literature, where NT has been identified as an important factor in nutrient metabolism particularly through lipid ingestion and fat storage [15, 16, 18–20]. Increased levels of peripheral NT occur in obesity and are directly linked to features of insulin resistance in humans [17, 19–21]. Li et al. were recently able to demonstrate a protection from obesity in NT-deficient mice fed with a high-fat diet [17], our findings provide additional support that dietary intervention may contribute to a reduction in pro-NT plasma levels.

### Limitations

Our study needs to be interpreted in light of its limitations. First, the study population was small, resulting in a restricted ability to draw definitive conclusions. Second, we had only one measurement of the biomarkers for the OMA-reference population. However, as a feasibility trial, LIBRE-1 aimed at generating hypotheses. Further investigation will be conducted in the framework of the larger LIBRE-2 trial with a sample size of more than 600 g*BRCA1/2* mutation carriers.

## Conclusion

Our study gives first indications that pro-NT, which is involved in tumorigenesis, cardiovascular- and obesity-related diseases, is inversely associated with improving cardiopulmonary fitness and nutritional parameters in g*BRCA1/2* mutation carriers. Yet, the situation was discrepant for pro-ENK, which showed a positive correlation with VO2peak at SE, however, further training did not lead to a corresponding increase in pro-ENK levels, possibly due to a type of “adrenal exhaustion”.

g*BRCA1/2* mutation carriers seem to have an adverse constellation of pro-NT and to a lesser extent pro-ENK, possibly due to intrinsic disposition.

Our findings support our supposition that improvements of lifestyle behavior including physical activity and dietary pattern, might constitute a strategy to reduce cancer risks, as well as mitigate cardiovascular risk in these mutation carriers.

## Data Availability

Individual participant data that underly the results reported in this article, will be available after deidentification (text, tables, figures). Moreover, the study protocol will be available. Data will be available beginning immediately following publication and ending 36 months following article publication. Data will be shared with researchers who provide a methodologically sound proposal to achieve aims in the approved proposal. There is no certain mechanism for data sharing applicable. Proposals should be sent to the corresponding author by e-mail.

## Abbreviations

BC: Breast cancer
BMI: Body Mass Index
CG: Control Group
CPET: Cardiopulmonary exercise testing
CV: Coefficient of variance
ENK: Enkephalin
FA: Fatty acid
g*BRCA1/2*: Germline mutation in the genes *BRCA1* and *BRCA2*
IG: Intervention Group
MedD: Mediterranean Diet
MET: Metabolic equivalent task
MetENK: Methionine-enkephalin
mRNA: Messanger ribnucleic acid
NT: Neurotensin
OC: Ovarian cancer
PBSO: Prophylactic bilateral salpingo-oophorectomy
pro-ENK: Pro-enkephalin
pro-NT: Pro-neurotensin
SE: Study Entry
V1: 3 Months after Study Entry in LIBRE-1
V2: 12 Months after Study Entry in LIBRE-1
VO2peak: Maximal oxygen uptake in ml/min/kg
